# Unicystic ameloblastoma of the mandible - an unusual case report and review of literature

**DOI:** 10.1186/1758-3284-2-1

**Published:** 2010-01-14

**Authors:** Rakesh S Ramesh, Suraj Manjunath, Tanveer H Ustad, Saira Pais, K Shivakumar

**Affiliations:** 1 Department of Surgical Oncology, St Johns Medical College Hospital, Sarjapur Road, Bangalore 560034, India

## Abstract

Ameloblastoma is a true neoplasm of odontogenic epithelial origin. It is the second most common odontogenic neoplasm, and only odontoma outnumbers it in reported frequency of occurrence. Its incidence, combined with its clinical behavior, makes ameloblastoma the most significant odontogenic neoplasm. Unicystic ameloblastoma (UA) refers to those cystic lesions that show clinical, radiographic, or gross features of a mandibular cyst, but on histologic examination show a typical ameloblastomatous epithelium lining part of the cyst cavity, with or without luminal and/or mural tumor growth. It accounts for 5-15% of all intraosseous ameloblastomas. We report a case of unicystic ameloblastoma in a 30-year-old female, and review the literature.

## Introduction

Many benign lesions cause mandibular swellings, and these can be divided into those of odontogenic and nonodontogenic origin. Lesions include ameloblastoma, radicular cyst, dentigerous cyst, keratocystic odontogenic tumour, central giant cell granuloma, fibro-osseous lesions and osteomas [1]. The most common tumour of odontogenic origin is ameloblastoma, which develops from epithelial cellular elements and dental tissues in their various phases of development. It is a slow-growing, persistent, and locally aggressive neoplasm of epithelial origin. Its peak incidence is in the 3rd to 4th decades of life and has an equal sex distribution. It is often associated with an unerupted third molar [2]. It may be detected during the course of routine radiography.

The vast majority of ameloblastomas arise in the mandible, and the majority of these are found in the angle and ramus region. There are three forms of ameloblastomas, namely multicystic, peripheral, and unicystic tumors [3]. Multicystic ameloblastoma is the most common variety and represents 86% of cases. Peripheral tumors are odontogenic tumors, with the histological characteristics of intraosseous ameloblastoma that occur solely in the soft tissues covering the tooth-bearing parts of the jaws. Unicystic tumors include those that have been variously referred to as mural ameloblastomas, luminal ameloblastomas, and ameloblastomas arising in dentigerous cysts [4]. The goal of treatment ameloblastoma is to achieve complete excision and appropriate reconstruction. We present a case of a large unicystic mandibular ameloblastoma in a 30 year old female.

## Case Report

**Figure 1:**
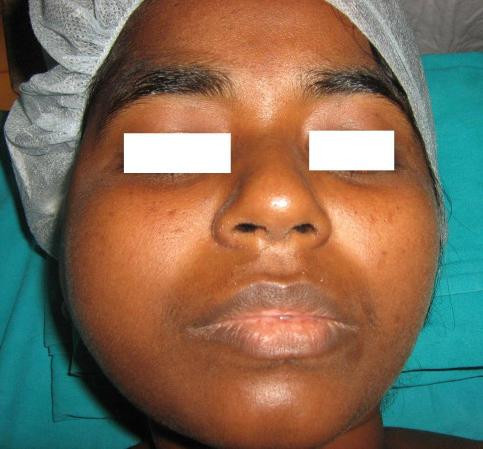
**Swelling right side of face**.

**Figure 2:**
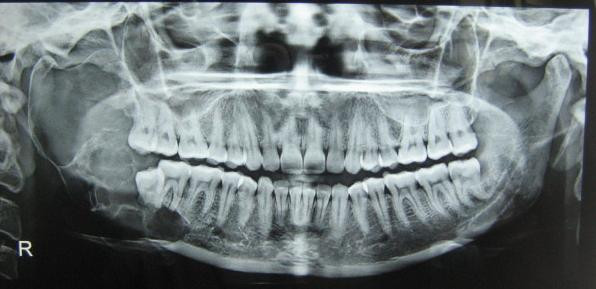
**OPG showing cystic lesion**.

**Figure 3:**
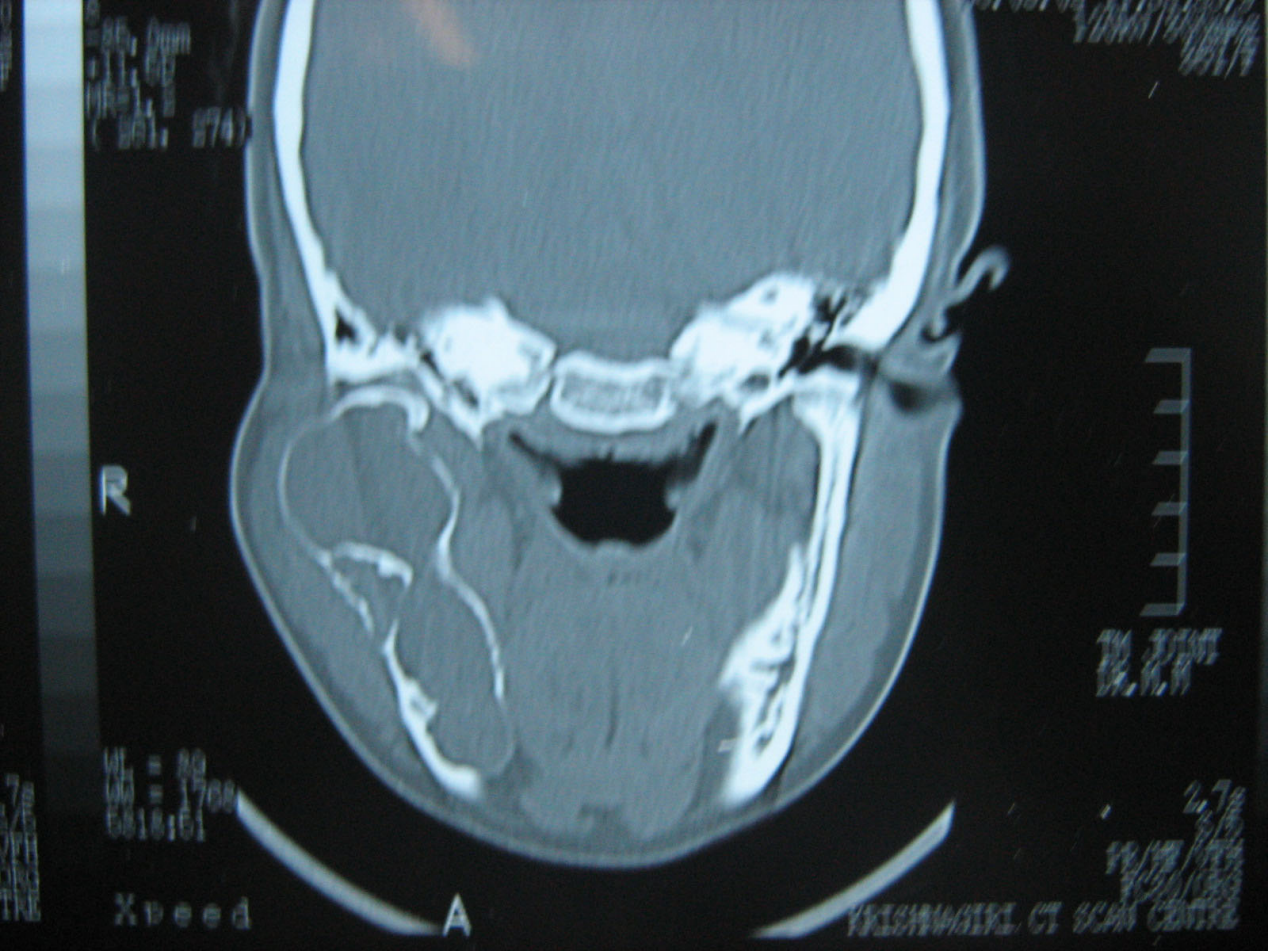
**CT scan showing lesion in right hemimandible**.

**Figure 4:**
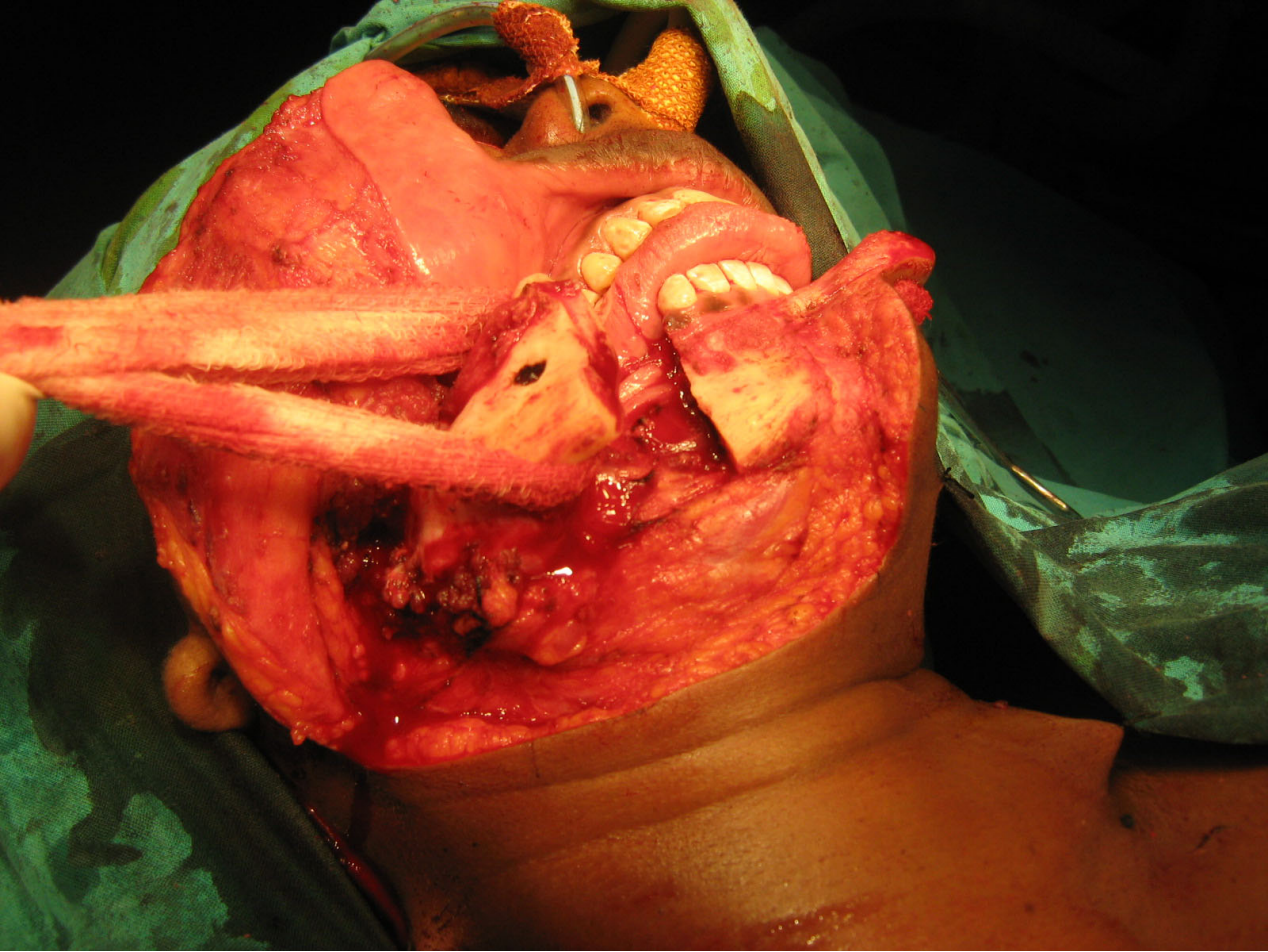
**Lip split approach - mandibotomy**.

**Figure 5:**
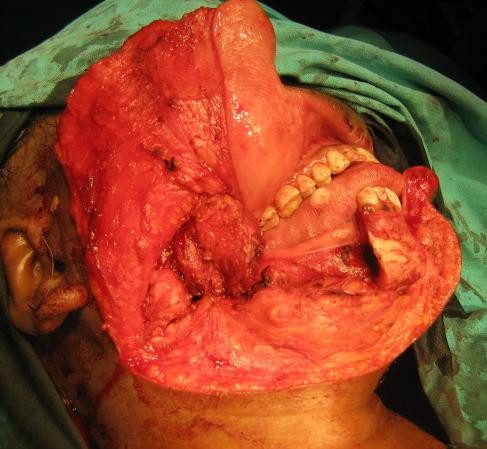
**Resection complete**.

**Figure 6:**
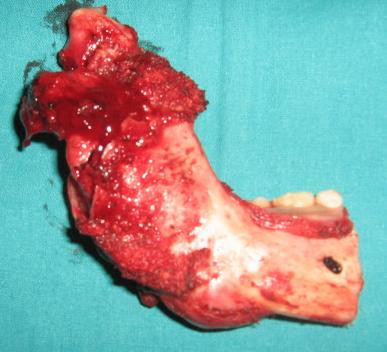
**Resected specimen**.

A 30 year old lady presented with a slowly growing swelling on the right side of the face since one year (Figure 1). There was no associated pain, difficulty in opening the mouth, chewing or articulating. On physical examination, there was a hard non-tender mass, measuring 8 cm by 5 cm arising from the right side of the mandible, involving the ramus, angle and body upto the right lower 1^st ^premolar tooth. The oral mucosa was normal. No neck nodes were palpable. Systemic examination was normal. An orthopantomogram (OPG) was done, which showed large cystic lesion in the right side of mandible (Figure 2). CT scan showed that the cystic lesion was confined to the mandible, with a thinned out cortex (Figure 3). The patient was taken up for surgery under general anaesthesia. A segmental mandibulectomy was done via a lip split incision (Figures 4, 5), and primary closure achieved. The resected specimen had histopathologic features consistent with unilocular ameloblastoma (Figure 6).

## Discussion

Unilocular ameloblastoma (UA) is a rare type of ameloblastoma, accounting for about 6% of ameloblastomas. It usually occurs in a younger age group, with about 50% of the cases occurring in the second decade of life. More than 90% are located in the mandible [5-7]. Between 50 and 80% of cases are associated with tooth impaction, the mandibular third molar being most often involved. The 'dentigerous' type occurs 8 years earlier on average than the 'non-dentigerous' variant. Patients most commonly present with swelling and facial asymmetry, pain being an occasional presenting symptom. Mucosal ulceration is rare, but may be caused by continued growth of the tumor. Small lesions are sometimes discovered more on routine radiographic screening examinations or as a result of local effects (like tooth mobility, occlusal alterations and failure of eruption of teeth) produced by the tumor [8]. Histologically, the minimum criterion for diagnosing a lesion as UA is the demonstration of a single cystic sac lined by odontogenic (ameloblastomatous) epithelium often seen only in focal areas. UA should be differentiated from odontogenic cysts because the former has a higher rate of recurrence than the latter [9]. In a clinicopathologic study of 57 cases of unicystic ameloblastoma, Ackermann [3] classified this entity into the following three histologic groups:

Group I: Luminal UA (tumor confined to the luminal surface of the cyst)

Group II: Intraluminal/plexiform UA (nodular proliferation into the lumen without infiltration of tumor cells into the connective tissue wall), and

Group III: Mural UA (invasive islands of ameloblastomatous epithelium in the connective tissue wall not involving the entire epithelium).

Another histologic subgrouping by Philipsen and Reichart [4] has also been described:

Subgroup 1: Luminal UA

Subgroup 1.2: Luminal and intraluminal

Subgroup 1.2.3: Luminal, intraluminal and intramural

Subgroup 1.3: Luminal and intramural

The UAs diagnosed as subgroups 1 and 1.2 can be treated conservatively (careful enucleation), whereas subgroups 1.2.3 and 1.3 showing intramural growths require treated radical resection, as for a solid or multicystic ameloblastoma [5]. Following enucleation, vigorous curettage of the bone should be avoided as it may implant foci of ameloblastoma more deeply into bone. Chemical cauterization with Carnoy's solution is also advocated for subgroups 1 and 1.2. Subgroups 1.2.3 and 1.3 have a high risk for recurrence, requiring more aggressive surgical procedures. This is because the cystic wall in these cases has islands of ameloblastoma tumor cells and there may be penetration into the surrounding cancellous bone [10-12]. Late recurrence following treatment is commonly seen, the average interval for recurrence being 7 years. Recurrence is also related to histologic subtypes of UA, with those invading the fibrous wall having a rate of 35.7%, but others only 6.7% [12]. Recurrence rates are also related to the type of initial treatment. Lau et al [13] reported recurrence rates of 3.6% for resection, 30.5% for enucleation alone, 16% for enucleation followed by Carnoy's solution application, and 18% by marsupialization followed by enucleation (where the lesion reduced in size).

## Conflict of interests

The authors declare that they have no competing interests.

## Authors' contributions

RSR participated in the surgical excision and drafted the manuscript

SM conceived the study and participated in drafting manuscript and co-ordination

THU obtained consent and photographs and participated in the literature search

SP participated in surgical excision and in drafting manuscript

SK performed the surgical excision and participated in literature search

All authors read and approved the final manuscript.

## Consent

Written informed consent was obtained from the patient for publication of this case report and accompanying images. A copy of the written consent is available for review by the Editor-in-Chief of this journal.
